# Acute Tubulointerstitial Nephritis: A Case Report on Rare Adverse Effect of Pembrolizumab

**DOI:** 10.3390/medicina55050176

**Published:** 2019-05-21

**Authors:** Sijan Basnet, Rashmi Dhital, Biswaraj Tharu

**Affiliations:** 1 Department of Internal Medicine, Reading Hospital, 420 S Fifth Avenue, West Reading, PA 19611, USA; rashmi.dhital@outlook.com; 2 Department of Internal Medicine, Trumbull Regional Medical Center, 1350 E Market St, Warren, OH 44483, USA; biswaraj.tharu@gmail.com

**Keywords:** pembrolizumab, acute kidney injury, programmed cell death 1 inhibitor, non-small cell lung cancer

## Abstract

Pembrolizumab is a novel immune checkpoint inhibitor approved for use in non-small cell lung carcinoma. There have been a few cases that have associated adverse renal outcomes with pembrolizumab. We present a case of acute kidney injury in a patient on pembrolizumab who was noted to have acute tubulointerstitial nephritis on renal biopsy. Pembrolizumab was discontinued and the patient was started on long-term corticosteroids with a taper. Her renal function improved partially with treatment.

## 1. Introduction

Pembrolizumab is an immune checkpoint inhibitor (CPI) approved by the United States Food and Drug Administration for use in non-small cell lung carcinoma (NSCLC) [1,2,3]. It prevents the suppression of T-cell activation by inhibiting binding of programmed cell death ligand (PD-L1) produced by tumor cells to the inhibitory programmed cell death (PD-1) receptor expressed on the surface of T-cells [1,2,4]. By doing this, T-cells can mediate an antitumor response. Pembrolizumab has been associated with side effects like fatigue, pruritus, and decreased appetite. Renal toxicity was not seen in initial reports [5]. Since then, renal adverse effects in association with CPIs have been described [2,4]. We present a case of NSCLC on pembrolizumab found to have acute kidney injury during hospitalization for chest and abdominal pain. Informed consent was obtained from the patient for submission of the case.

## 2. Case Description

The patient is a 62-year-old woman who was brought to the emergency department (ED) with 2 episodes of sudden onset substernal chest pain, each episode lasting for 30 min. Her chest pain had resolved at the time of arrival. Prior to that, she had felt nauseous which was usual for her after her chemotherapy. Chest pain was followed by right-sided, sharp diffuse abdominal pain which lasted for 10 min and resolved spontaneously. She had received her last chemotherapy infusion 2 days prior to the episode. She denied any fever, chills, cough or shortness of breath. She was diagnosed of NSCLC with bone metastases (epidermal growth factor receptor negative and PD-L1-80%) a year ago for which she underwent radiation therapy of left hip and right upper ribs, completed palliative chemotherapy with 6 cycles of pemetrexed 500 mg/m^2^/dose, carboplatin 550 mg, and pembrolizumab 200 mg followed by same doses of pemetrexed and pembrolizumab maintenance every 3 weeks with last dose 2 days prior to presentation. The patient had been on pembrolizumab for 6 months prior to the decline in renal function. Other past medical history included stage IA right breast cancer (estrogen receptor+ (90%), progesterone receptor+ (3–5%), and human epidermal growth factor receptor 2-negative invasive ductal carcinoma) for which she underwent a bilateral mastectomy, 6 cycles of cyclophosphamide, methotrexate, and fluorouracil, and tamoxifen for 5 years 20 years ago, hypothyroidism, and hyperlipidemia. Her home medications included levothyroxine 75 µg daily, folic acid 1 mg daily, pantoprazole 40 mg daily, rosuvastatin 5 mg nightly, dexamethasone 8 mg two doses before and after chemotherapy, olanzapine 10 mg nightly, lorazepam 0.5 mg as needed, ondansetron 8 mg as needed, prochlorperazine 10 mg as needed, and promethazine 25 mg as needed. She had smoked a pack a day for 15 years before quitting 27 years ago.

On examination, vitals were stable with a temperature of 36.7 °C (98.1 °F), blood pressure 139/82 mm Hg, pulse 79 beats per minute, respiratory rate 18 breaths per minute, and she was maintaining saturation on room air. Chest, cardiac, and abdominal examinations were unremarkable. Her hemoglobin was 9.1 g/dL (reference range: 12.0–16.0 g/dL), platelet count was 556,000/µL (reference range: 13,000–400,000/µL), and white count was 10,700/µL (reference range: 4800–10,800/µL) with neutrophil count of 9800/µL (reference range: 2000–8000/µL), monocyte count of 400 (reference range: 100–1300/µL), and immature granulocyte count of 260/µL (reference range: 0–30/µL). Serial troponins and electrocardiograms were non-suggestive of an acute coronary syndrome. Her creatinine was 1.69 mg/dL (reference range: 0.6–1.3 mg/dL). It was 1.72 two days prior above her baseline of 0.6–0.9. Although d-dimer was 1.02 (reference range: <0.53 µg/mL), computed tomography (CT) pulmonary embolism protocol was not done as ultrasound lower extremity vein bilateral was negative and there was low clinical suspicion for pulmonary embolism. CT without contrast of chest/abdomen/pelvis showed decreasing right upper lobe mass and surrounding consolidation. She had bilateral enlarging metastatic lung nodules. Hepatic metastases were not identified but contrast was not used. Bone metastases were unchanged. Her kidneys and urinary tract on CT and urinalysis were unremarkable. Her acute kidney injury (AKI) was thought to be related to poor oral intake and vomiting related volume depletion. The patient was discharged home and was recommended hydration and repeat labs in a few days. Her creatinine on the day of discharge was 1.83 mg/dL. 

**Figure 1:**
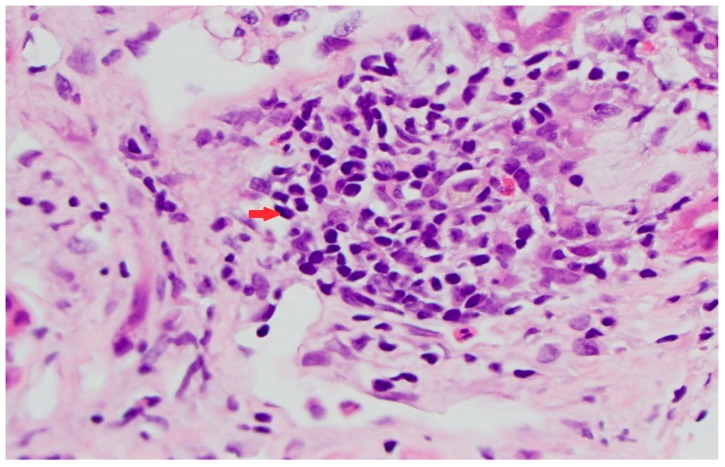
Renal biopsy with focal interstitial inflammation, mostly lymphocytes (arrow) and focal tubulitis.

Two days post discharge, the patient called her oncologist and told that she had started feeling sick on the same day after she was discharged. She complained of fever with maximum recorded temperature was 102.7 °F along with chills and rigors. She had multiple episodes of vomiting and poor intake. She was directly admitted to the hospital. At presentation, her vitals were stable with a temperature of 37.0 °C (98.6 °F), blood pressure 140/71 mm Hg, pulse 84 beats per minute, and respiratory rate 20 breaths per minute. Chest, cardiac, and abdominal examinations were unremarkable. Her mucous membranes were moist. Her hemoglobin was 8.2 g/dL (reference range: 12.0–16.0 g/dL), platelet count was 257,000/µL (reference range: 13,000–400,000/µL), white count was 2600/µL (reference range: 4800–10,800/µL) with neutrophil count of 1500/µL (reference range: 2000–8000/µL), monocyte count of 0/µL (reference range: 100–1300/µL), and immature granulocyte count of 50/µL (reference range: 0–30/µL). Her low cell counts were thought to be related to her chemotherapy. No eosinophilia was noted in complete blood count during hospitalization or prior to presentation. Her urinalysis was negative for infection but showed 30 mg /dL (1+) proteinuria. Her blood and urine cultures showed no growth. Her creatinine was elevated at 3.70 mg/dL and this was again thought to be related to volume depletion. The patient was started on intravenous fluids. Her renal ultrasound was unremarkable. With suspicion for pembrolizumab as a potential cause for acute kidney injury, future infusions were held. The patient was given a dose of IV methylprednisone 80 mg (1 mg/kg) and planned to be started on prednisone 80 mg daily next day. She was planned for a renal biopsy to rule out medication-related injury. Her creatinine progressively improved during her 5-day-stay and was 2.10 on discharge. We think this was with discontinuation of pembrolizumab. Her renal biopsy showed evidence of acute tubular injury, focal interstitial inflammation (lymphocytes, plasma cells, few eosinophils, few neutrophils) with focal mild tubulitis, 14% globally sclerotic glomeruli, mild arterial thickening, and mild interstitial fibrosis (Figure 1). This was thought to be secondary to pembrolizumab which was permanently discontinued. She was started on docetaxel 125 mg every 3 weeks. She has received 3 cycles so far. With non-improvement in kidney function, prednisone dose was increased to 1 mg/kg/day (70 mg) for a course of 3 months. Sulfamethoxazole-trimethoprim and pantoprazole were started for prophylaxis. Her creatinine even after 5 months is still elevated. Her new creatinine baseline is around 1.8–2.0. Pertinent case details are summarized in Table 1. 

## 3. Discussion

Renal adverse effects are rare with CPIs [6]. The overall incidence of acute kidney injury was reported to be 2.2% among 3695 patients on a CPI by Cortazar et al. [4] and 1.77% among 676 pembrolizumab by Izzedine et al. [6]. Acute kidney injury can occur at any time, from 6 to 10.5 weeks up to 24 months after treatment initiation [4,6]. It may take 3–6 weeks for resolution [6]. No gender predisposition has been noted [2]. Two different patterns of renal parenchymal damage have been noted on renal biopsy, acute (granulomatous) tubulointerstitial nephritis (ATIN) and immune complex glomerulonephritis [6]. ATIN was reported in 4 out of 12 [6], 12 out of 13 [4], and 14 out of 16 [7] with AKI. ATIN is thought to result from loss of tolerance of self-reactive T-cells against renal antigens by inhibition of inhibitory the PD-1/PD-L1 signaling pathway [2,4,6]. This triggers an inflammatory response against renal parenchymal tissue manifesting as ATIN [2,6]. Shirali et al. report 6 cases of acute interstitial nephritis in association with CPIs. Out of them, 3 were on proton pump inhibitors (PPIs) like our patient. They theorize that initiation of PD-1 inhibitor therapy may have disrupted tolerance to PPIs and precipitated ATIN [8]. Our patient was on pantoprazole for years for subjective acid reflux which was continued during and after pembrolizumab was stopped. It might have contributed to the worsened renal injury. It was continued for gastrointestinal protection as the patient was placed on long term prednisone. 

Renal biopsy is recommended in cases where AKI from pembrolizumab is suspected. Discontinuation of pembrolizumab and initiation of corticosteroids is the mainstay of treatment [2,6,8]. Prognosis is excellent with corticosteroids [4,6]. Among the 12 patients with ATIN, 10 patients who received glucocorticoids had partial or complete recovery of the renal function while 2 had non-improvement in the absence of glucocorticoids [4]. Similar outcome was noted in 6 alive pembrolizumab-treated patients who had a recovery of ~50% of their renal function [6]. Similarly, Shirali et al. reported 6 biopsy-proven cases of interstitial nephritis with checkpoint inhibitors that improved with discontinuation of the medication and course of corticosteroids [8]. Izzedine et al. recommend careful corticosteroid taper over a month [6]. Mamlouk et al. used prednisone at doses ranging between 0.5–4 mg/kg/day which was tapered off 4–24 months based on the pathology and recurrence of renal disease [7]. Longer courses and additional immunosuppressive medications may be needed in patients with poor response [6]. Infliximab has been tried to improve renal function [7]. Re-challenge with PD-1 inhibitor therapy can be considered with improvement in renal function and withdrawal of any other possible offending agents [6,8]. Recurrence of severe ATIN resulted with reintroduction of pembrolizumab. Our patient’s renal function never recovered and she progressed to chronic kidney disease stage IV from stage II. Hence, further treatment with pembrolizumab was aborted.

## 4. Conclusion

Clinicians should be aware of the potential renal complications of pembrolizumab which can lead to a break in the treatment of the underlying condition. Early identification of an increase in creatinine with regular monitoring may help with prevention and early institution of effective treatment. 

## Figures and Tables

**Table 1:** Summary of case details.

S.N.	Headings	Prior to Hospitalization	First Hospitalization	Index Hospitalization
1.	Serum creatinine (reference range: 0.6–1.3 mg/dL)	<1 mg/dL	Day 1: 1.69 mg/dL Discharge: 1.83 mg/dL	Day 1: 3.70 mg/dL (also peak) Discharge: 2.10 mg/dL
2.	Estimated GFR (mL/min/1.73 m^2^)	>70 mL/min/1.73 m^2^	Day1: 30.66 mL/min/1.73 m^2^ Discharge: 27.97 mL/min/1.73 m^2^	Day 1: 12.41 mL/min/1.73 m^2^ Discharge: 23.86 mL/min/1.73 m^2^
3.	Presentation		Substernal chest pain, diffuse abdominal pain	Fever and vomiting
4.	Workup		Normal kidneys and urinary tract on computed tomography Normal urinalysis	Normal renal ultrasound and urinalysis
5.	Treatment		Intravenous hydration	Intravenous hydration Pembrolizumab permanently discontinued Intravenous methylprednisone 80 mg (1 mg/kg) followed by prednisone for 3 months and 3 cycles of docetaxel 125 mg every 3 weeks. Sulfamethoxazole-trimethoprim and pantoprazole for prophylaxis.
